# Tazobactam/ceftolozane and tobramycin combination therapy in extensively drug-resistant Pseudomonas aeruginosa infections in severe burn injury: a case report

**DOI:** 10.1186/s40780-023-00294-x

**Published:** 2023-08-08

**Authors:** Yuta Ibe, Ryuichiro Kakizaki, Hirotoshi Inamura, Tomoyuki Ishigo, Yoshihiro Fujiya, Hiroyuki Inoue, Shuji Uemura, Satoshi Fujii, Satoshi Takahashi, Eichi Narimatsu, Masahide Fukudo

**Affiliations:** 1 Department of Hospital Pharmacy, Sapporo Medical University Hospital, South-1, West-16, Chuo-Ku, Sapporo, 060-8543 Japan; 2 Department of Emergency Medicine, Sapporo Medical University School of Medicine, Sapporo, Japan; 3 Department of Infection Control and Laboratory Medicine, Sapporo Medical University School of Medicine, Sapporo, Japan

**Keywords:** Burns injury, *Pseudomonas aeruginosa*, Tazobactam/ceftolozane, Tobramycin

## Abstract

**Background:**

Combination therapy with tazobactam/ceftolozane (TAZ/CTLZ) and high-dose aminoglycosides has been reported to be efficacious in extensively drug-resistant (XDR)-*Pseudomonas aeruginosa* infection. However, there are no reports of efficacy in XDR-*P. aeruginosa* infection for combination therapy with low-dose aminoglycosides and TAZ/CTLZ. Herein, we describe a rare case of severe burn injury patients with persistent bacteremia due to XDR-*P. aeruginosa*, which was successfully treated with TAZ/CTLZ and low-dose tobramycin (TOB).

**Case presentation:**

A 31-year-old man was admitted to the intensive care unit with severe burn injury involving 52% of the total body surface area and a prognostic burn index of 79.5. The patient had recurrent bacterial infections since admission, and blood cultures collected on the 37th day of admission revealed the presence of *P. aeruginosa* strains that were resistant to all β-lactams and amikacin (AMK). The results of the antimicrobial synergistic study showed no synergistic effect of low-dose meropenem (MEPM) and AMK combination therapy. The patient had acute renal failure, and it was difficult to increase the dose of MEPM and AMK, respectively. Thus, we initiated TAZ/CTLZ 1.5 g/8 h instead of the AMK and MEPM combination therapy on the 43rd day of hospitalization. Low-dose TAZ/CTLZ was continued because of prolonged renal dysfunction and resulted in a transient clinical improvement. However, the dosage of TAZ/CTLZ could be increased as the renal function improved, but despite an increased TAZ/CTLZ dose, bacteremia persisted, and the blood cultures remained positive. Thus, TOB was added to TAZ/CTLZ at low doses for synergistic effect against Gram-negative bacteria. Blood cultures collected after initiation of combination therapy with TAZ/CTLZ and low-dose TOB were negative on two consecutive follow-up evaluations. Thereafter, although the patient had several episodes of fever and increased inflammatory response, blood cultures consistently tested negative, and all of the wounds healed. On the 93rd day, due to the good healing progress, the patient was transferred to another hospital.

**Conclusions:**

TAZ/CTLZ and low-dose TOB combination therapy showed the potential for synergistic effects. Our present report suggests a novel synergistic treatment strategy for rare cases that are refractory to the treatment of infections, such as XDR-*P. aeruginosa* infection.

**Supplementary Information:**

The online version contains supplementary material available at 10.1186/s40780-023-00294-x.

## Introduction

Burn patients in the out of acute-phase of their injury experience a range of clinical problems [1], with one key factor being the higher prevalence of drug-resistant bacteria [2], which further complicates their treatment. In particular, *Pseudomonas aeruginosa* causes systemic infections through wound infections and is associated with an increased mortality in burn care units [3].

Tazobactam/ceftolozane (TAZ/CTLZ) is a novel antibacterial drug combination, comprising a cephalosporin (ceftolozane) and a beta (β)-lactamase inhibitor (tazobactam) [4]. This drug is effective against *P. aeruginosa* [5]; however, TAZ/CTLZ is not effective against carbapenemase-producing strains [6] but is effective against AmpC- and extended-spectrum β-lactamase-producing strains. TAZ/CTLZ has demonstrated high activity against *P. aeruginosa*, in vitro [7] and is expected to be effective against 90% of primary β-lactam-resistant strains [8]. Therefore, TAZ/CTLZ might be effective against multidrug-resistant (MDR) and extensively drug-resistant (XDR)-*P. aeruginosa* isolates. However, MDR- and XDR-*P. aeruginosa* strains have decreased susceptibility to β-lactams, quinolones, and aminoglycosides, and monotherapy with any antibiotic has limited efficacy. Notably, combination therapy with β-lactams and aminoglycosides or quinolones may be considered empirical for the treatment of severe *P. aeruginosa* infections [9]. High-dose aminoglycoside antibiotics have been shown to be effective against XDR-*P. aeruginosa* in combination therapy with TAZ/CTLZ [10, 11]; however, to our knowledge, there are no reports of efficacy for combination therapy with low-dose aminoglycosides used synergistically with TAZ/CTLZ.

Herein, we describe the first case of severe burn injury with persistent bacteremia due to XDR-*P. aeruginosa*, which was successfully treated with a combination of TAZ/CTLZ and low-dose tobramycin (TOB).

## Case presentation

A 31-year-old man was admitted to the intensive care unit with severe burn injury involving 52% of the total body surface area and a prognostic burn index of 79.5. The clinical data recorded at the time of hospitalization are presented in Table S1. After hospitalization, an escharotomy was immediately performed, and fluid resuscitation and mechanical ventilatory management were initiated. Devices, such as central venous catheter, arterial line, and flexible double lumen catheter, were inserted. The patient developed acute kidney injury (AKI) and received continuous renal replacement therapy (CRRT) immediately after hospitalization.

The first surgical debridement was performed on the 3rd day of hospitalization. On the fourth day of hospitalization, the patient developed fever and decreased blood pressure. Meropenem (MEPM) and linezolid (LZD) were initiated as treatment for septic shock. Blood and wound culture results revealed the presence of *Klebsiella species*, *Serratia marcescens*, *Enterobacter cloacae complex*, and several other indigenous skin bacteria. Based on the susceptibilities of the bacterial species that were identified, MEPM and LZD were replaced with sulbactam/ampicillin (SBT/ABPC) and ceftazidime (CAZ) to target bacteria, excluding indigenous skin bacteria. Subsequently, the surgical debridement was performed on the 5th and 10th days of hospitalization. Since the clinical course was favorable and blood samples collected and cultured at the follow-up evaluation were negative for the target bacteria, both SBT/ABPC and CAZ administration were discontinued after the 5-day regimen.

**Fig. 1:**
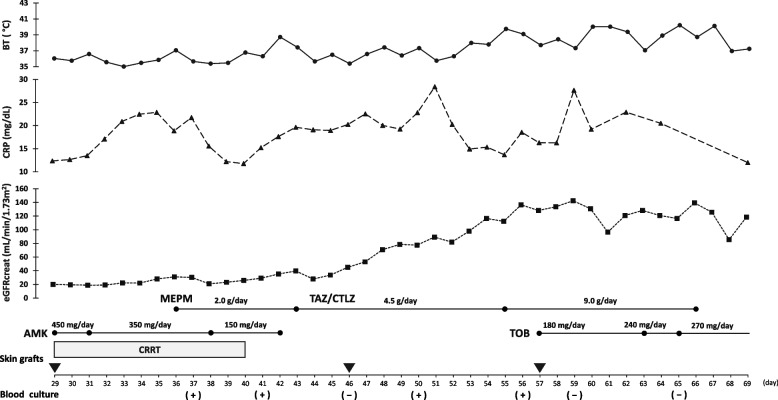
Clinical course of this case. Abbreviations: AMK, amikacin; BT, body temperature; CRP, C-reactive protein; TAZ/CTLZ, tazobactam/ceftolozane; MEPM, meropenem; TOB, tobramycin

**Fig. 2:**
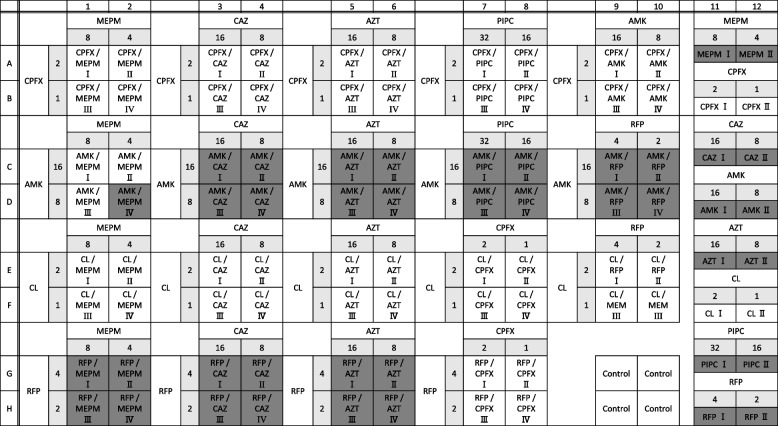
Antimicrobial Synergy Study – Checkerboard Testing Abbreviations: AMK, amikacin; AZT, aztreonam; CAZ, ceftazidime; CPFX, ciprofloxacin; CL, colistin; MEPM, meropenem; PIPC, piperacillin; RFP, rifampicin. Representation of a checkerboard assay where the synergistic effect of two antibiotics is depicted. In this illustration, “no growth” is represented in white, and “growth” is represented in black

The second skin graft surgery was performed on the 29th day after admission; carbapenem-resistant *P. aeruginosa* was detected for the first time in the burn wounds and in sputum cultures collected before grafting (Table 1). Amikacin (AMK) monotherapy was selected as a perioperative therapeutic strategy for burn wounds (Fig. 1). However, because the patient was receiving CRRT support for AKI due to burns, the blood levels of AMK did not reach the target peak concentration required for therapeutic effect. Furthermore, trough concentrations remained high; therefore, the dose was reduced and continued. AMK blood concentration results are shown in Table S4. Thereafter, a chest X-ray showed partial loss of permeability in the right lung field, and an increased inflammatory response was confirmed, possibly due to postoperative ventilator management, suggesting the possibility of ventilator-associated pneumonia. As AMK diffuses poorly in the lung tissue, MEPM was included as a treatment for pneumonia caused by *P. aeruginosa*, and the ventilator was weaned off. However, the blood sample collected at the follow-up evaluation revealed β-lactams and AMK-resistant *P. aeruginosa.* The drug susceptibility results of *P. aeruginosa* detected in blood cultures (blood culture samples at days 37, 41, 50, and 56) are shown in Table 1. The antimicrobial synergistic study of *P. aeruginosa* was conducted using the Eiken breakpoint checkerboard plate (BC plate®) (Fig. 2). The results were analyzed using the interpretive criteria defined by the Clinical and Laboratory Standards Institute. Notably, the low-dose MEPM and AMK combination therapy did not exhibit a synergistic effect. Due to the patient’s AKI complications, it was difficult to increase the MEPM and AMK doses.

**Table 1:** Antibiotic sensitivity test results of the isolated *Pseudomonas aeruginosa*

Antibiotics	day 27			day 37			day 41			day 50			day 56		
	Burn wounds / sputum cultures			Blood culture			Blood culture			Blood culture			Blood culture		
	MIC (μg/dL)		Interpretation	MIC (μg/dL)		Interpretation	MIC (μg/dL)		Interpretation	MIC (μg/dL)		Interpretation	MIC (μg/dL)		Interpretation
Piperacillin	>	64	R	>	64	R	>	64	R	>	64	R	>	64	R
Tazobactam/piperacillin	>	64	R	>	64	R	>	64	R	>	64	R	>	64	R
Ceftazidime	>	16	R	>	16	R	>	16	R	>	16	R	>	16	R
Cefepime	>	16	R	>	16	R	>	16	R	>	16	R	>	16	R
Aztreonam	>	16	R	>	16	R	>	16	R	>	16	R	>	16	R
Imipenem/cilastatin	>	8	R	>	8	R	>	8	R	>	8	R	>	8	R
Meropenem	>	8	R	>	8	R	>	8	R	>	8	R	>	8	R
Amikacin	=	8	S	>	32	R	>	32	R	=	32	I	=	32	I
Gentamicin	=	4	S	=	4	S	>	8	R	=	8	I	=	8	I
Tobramycin	≤	4	S	≤	4	S	≤	4	S	≤	4	S	≤	4	S
Colistin	≤	2	S	≤	2	S	≤	2	S	≤	2	S	≤	2	S
Levofloxacin	≤	0.5	S	=	2	S	≤	0.5	S	=	2	S	=	2	S
Ciprofloxacin	≤	1	S	=	1	S	≤	0.25	S	=	0.5	S	=	0.5	S

Abbreviations: *MIC* Minimum inhibitory concentration, *R* Resistant, *S* Sensitive, *I* Intermediate

Therefore, we initiated TAZ/CTLZ 1.5 g/8 h instead of the AMK and MEPM combination therapy on the 43rd day of hospitalization (Fig. 1). Although the antimicrobial susceptibility test of TAZ/CTLZ could not be performed on the day of hospitalization, the results of the genetic analysis confirmed that *P. aeruginosa* that was isolated was a non-carbapenemase-producing strain. The patient could be weaned off from CRRT on the 40th day of hospitalization, but low-dose TAZ/CTLZ was continued because of prolonged renal dysfunction, which resulted in a transient clinical improvement. The third skin graft surgery was performed on the 46th day of hospitalization. However, fever and an increased inflammatory response were observed, and XDR-*P. aeruginosa* was detected on the blood culture as in previous blood cultures. The treatment could have failed due to underdosing with TAZ/CTLZ or bacterial resistance to TAZ/CTLZ at that time. Therefore, the TAZ/CTLZ dose was increased to 3 g/8 h in accordance with the renal function.

The fourth skin graft surgery was performed on the 57th day of hospitalization. However, despite an increased TAZ/CTLZ dose, bacteremia persisted, and the blood cultures remained positive. Because of the limited therapeutic effect of TAZ/CTLZ monotherapy, the TOB susceptible to this isolate (180 mg/24 h) was initiated. TOB was added on TAZ/CTLZ at low doses for synergistic effect against Gram-negative bacteria. The therapeutic drug monitoring results of TOB are shown in Table S2. Blood cultures collected after initiation of combination therapy with TAZ/CTLZ and TOB were negative on two consecutive follow-up evaluations. Since the blood cultures were negative, the patient’s persistent bacteremia was considered to be under control. Moreover, since *P. aeruginosa* may become resistant to TAZ/CTLZ with long-term use, a higher dose of TOB was considered in order to switch to standard therapy for Gram-negative bacteria. In addition, since the clinical course was favorable, TOB was increased as standard monotherapy against Gram-negative bacteria, and the peak concentration measured on the 69th day of hospitalization reached the therapeutic response range. Combination therapy with TAZ/CTLZ and TOB was continued for 10 days, and then treatment with TOB as a monotherapy was continued for the remainder of the treatment period, which was set at 14 days after negative blood cultures. Thereafter, although the patient had several episodes of fever and increased inflammatory response, blood cultures consistently tested negative, and all of the wounds healed. On the 93rd day of hospitalization, due to the good healing progress, the patient was transferred to another hospital.

Although susceptibility testing of TAZ/CTLZ was not possible, we performed retrospective antimicrobial susceptibility testing of TAZ/CTLZ on *P. aeruginosa* isolated from blood cultures as an additional analysis (Table S3). The results from samples collected before TAZ/CTLZ administration showed that one of the isolates was resistant, while another showed intermediate susceptibility.

## Discussion

This report describes a significant clinical case where TAZ/CTLZ and low-dose TOB combination therapy was successfully used to treat XDR-*P. aeruginosa* infection in a patient with severe burns. To our knowledge, this treatment approach has not been previously reported.

In patients with severe burns, infection is the most common complication that is associated with an increased mortality risk [3]. The burn wound is initially colonized by a higher proportion of Gram-positive bacteria with continued antimicrobial therapy, these bacteria are often replaced by Gram-negative ones [12]. A 50% increase in mortality has been reported in patients with burns having Gram-negative bacteremia compared with those without bacteremia [13]. Moreover, due to the increasing antibiotic resistance, the treatment of infections has become difficult and has contributed to the increased mortality rate. Furthermore, MDR-*P. aeruginosa* causes 4–60% of nosocomial infections, which leads to high mortality and morbidity in patients with burns [14, 15]. Therefore, controlling *P. aeruginosa* and preventing the development of antibiotic-resistant strains is crucial for infection control in burn wards and successful treatment of burns. In this case, XDR-*P. aeruginosa* was detected within 1 month after the first positive blood culture, and bacteremia caused by this isolate persisted; thus, controlling XDR-*P. aeruginosa* was necessary for the successful treatment of this patient.

Combination therapy with more than two antibiotics with anti-*P. aeruginosa* activity is recommended for the treatment of severe *P. aeruginosa* infections to decrease the risk of treatment failure. Among the effective regimens, the β-lactam and aminoglycoside combination is frequently used, and this combination is effective against MDR-*P. aeruginosa* infections [16, 17]. In this case, the MEPM and AMK combination therapy used to treat XDR-*P. aeruginosa* infection did not result in any clinical improvement. A possible cause of treatment failure was that this patient had AKI and was receiving low doses of MEPM and AMK. In support of this, a synergistic study of antibacterial activity against resistant *P. aeruginosa* showed that the combination of MEPM and AMK at low doses did not show a synergistic effect (Fig. 2).

A recent study reported that TAZ/CTLZ therapy is more effective than colistin, polymyxin, or aminoglycoside-based regimens, in severe *P. aeruginosa* infections. The CEFTABUSE registry results showed that the 14-day clinical cure rates of TAZ/CTLZ administration were more effective than those of aminoglycosides and polymyxin, although the difference was not statistically significant [18]. A retrospective cohort study showed that TAZ/CTLZ administration was independently associated with clinical cure compared with colistin- or aminoglycoside-based regimens [19]. These two studies indicated that the incidence of AKI was significantly lower with the TAZ/CTLZ treatment group, indicating that TAZ/CTLZ can be an effective option for patients with renal dysfunction or those with a high risk of nephrotoxicity. However, in this case, treatment with TAZ/CTLZ monotherapy failed to suppress the persistent bacteremia caused by resistant *P. aeruginosa*. This could be due to the fact that retrospective *P. aeruginosa* drug susceptibility testing had revealed a TAZ/CTLZ-resistant strain.

In our case, persistent bacteremia caused by resistant *P. aeruginosa* was controlled after initiation of combination therapy with TAZ/CTLZ and low-dose TOB. We believe that the fact that blood cultures became negative on two consecutive follow-up evaluations after the start of combination therapy with TAZ/CTLZ and TOB proves the effectiveness of the combination therapy. The success of this combination therapy supported two previous reports [10, 11].　An in vitro study has assessed the efficacy of a combination therapy of TAZ/CTLZ and an aminoglycoside [10], demonstrating that the TAZ/CTLZ and AMK combination therapy has synergistic effects and may therefore be useful in treating MDR-*P. aeruginosa* infections. In another study conducted on febrile neutropenic patients with concurrent TAZ/CTLZ -resistant *P. aeruginosa* infection, the combination of TAZ/CTLZ and TOB synergistically decreased the respective minimal inhibitory concentration values [11]. Thus, in our case, the addition of TOB improved the anti-microbial effects and possibly enhanced the efficacy of TAZ/CTLZ. One difference between these two previous reports and the present case is the dosage of TOB. In these two previous studies, the dosing settings were 25 mg/kg/q24h for AMK and 7 mg/kg/q24h for TOB, which were capable of reaching the target peak concentrations required for therapeutic effects. In our case, TOB was initiated at a dose of 3 mg/kg, which is much lower than the dose required to achieve the target peak concentration to produce a therapeutic effect (5–7 mg/kg/q24h). Furthermore, renal function at the time of TOB initiation in this patient was eGFRcreat > 130 mL/min/1.73 m^2^, a condition suspicious for augmented renal clearance (ARC). Previous reports suggest that the recommended dose of TOB in patients with ARC is 7 mg/kg/day to achieve pharmacodynamic goals [20]. It has also been reported that split doses or higher doses may be required to reach target peak concentrations in severe burn patients with increased volume of distribution and increased clearance [21, 22]. Generally, when TOB is administered at a low dose for its synergistic effects against Gram-negative bacteria, its peak concentration is not routinely measured. However, actual measured TOB trough concentrations (Table S2) and patient factors also suggest that the effective peak concentration has not been reached. Despite a low dose of TOB for persistent bacteremia, the success in achieving negative blood culture results suggests an enhanced synergistic efficacy of the TAZ/CTLZ and TOB combination therapy.

We did not select quinolones as an antibiotic treatment option for XDR-*P. aeruginosa*. Although quinolones constitute effective therapeutic options for *P. aeruginosa* infections, a meta-analysis indicated that the use of quinolones increased the risk of MDR- or XDR-*P. aeruginosa* compared with that of resistant or susceptible *P. aeruginosa* [23]. Therefore, the use of quinolones was avoided here to prevent *P. aeruginosa* from acquiring multidrug or extensive drug resistance.

In our case, TAZ/CTLZ susceptibility testing of *P. aeruginosa*-resistant strains was performed retrospectively, which has rarely been reported of *P. aeruginosa* strains that acquired resistance prior to TAZ/CTLZ exposure in Japan. The stability of TAZ/CTLZ against the main resistance mechanisms of *P. aeruginosa,* such as OprD deficiency, increased AmpC production, and drug excretion proteins [6, 24], underscores that TAZ/CTLZ resistance can result from the intense use of other β-lactam antibiotics that can induce or inhibit AmpC production, independently of TAZ/CTLZ use [25]. Therefore, it is possible that the use of multiple β-lactam antibiotics during the 1-month burn treatment period contributed to the development of TAZ/CTLZ resistance. However, the mechanisms driving TAZ/CTLZ resistance need to be further explored. Notably, the combination of TAZ/CTLZ and low-dose aminoglycoside antibiotics may be an effective antimicrobial treatment option, even in *P. aeruginosa* strains that exhibit TAZ/CTLZ resistance.

A limitation of this study is that no antimicrobial synergism study of TAZ/CTLZ and TOB was conducted; therefore, TOB monotherapy may have been more effective than TAZ/CTLZ and TOB synergistic therapy. Therefore, future antimicrobial synergistic studies of TAZ/CTLZ and low-dose aminoglycosides in vitro should be conducted to clearly distinguish between additive and synergistic effects. However, we consider it a clinically serious finding that in the present case, treatment with TAZ/CTLZ and low-dose TOB resulted in negative blood cultures for *XDR-P. aeruginosa* bacteremia resistant to TAZ/CTLZ.

In conclusion, this case highlights the efficacy of a combination therapy with TAZ/CTLZ and low-dose TOB in managing persistent XDR-*P. aeruginosa* bacteremia in patients with severe burns. Furthermore, the combination of TAZ/CTLZ with low-dose TOB is a promising antimicrobial option for TAZ/CTLZ-resistant *P. aeruginosa* infections. Further research is warranted to validate the findings of this study and explore the potential benefits and limitations of this regimen.

## Supplementary Information

**Additional file 1: Table S1**. Laboratory data. **Table S2.** Blood concentration level of tobramycin. **Table S3.** Antimicrobial susceptibility testing of tazobactam/ceftolozane against *Pseudomonas aeruginosa. ***Table S4.** Blood concentration level of amikacin.

## Data Availability

Not applicable.

## Abbreviations

TAZ/CTLZ: Tazobactam/ceftolozane
MDR: Multidrug-resistant
XDR: Extensively drug-resistant
TOB: Tobramycin
AKI: Acute kidney injury
CRRT: Continuous renal replacement therapy
MEPM: Meropenem
LZD: Linezolid
SBT/ABPC: Sulbactam/ampicillin
CAZ: Ceftazidime
AMK: Amikacin
ARC: Augmented renal clearance
