# Supplementary material for: Tazobactam/ceftolozane and tobramycin combination therapy in extensively drug-resistant Pseudomonas aeruginosa infections in severe burn injury: a case report
Source: J Pharm Health Care Sci. 2023 Aug 8;9:25. doi: 10.1186/s40780-023-00294-x (PMC10408163; doi:10.1186/s40780-023-00294-x)
Supplement: Supplementary file 1 — Additional file 1: Table S1. Laboratory data. Table S2. Blood concentration level of tobramycin. Table S3. Antimicrobial susceptibility testing of tazobactam/ceftolozane against Pseudomonas aeruginosa. Table S4. Blood concentration level of amikacin. [file 40780_2023_294_MOESM1_ESM.docx]

**Table S1. Laboratory data**

|  | Day 1 ^1)^ |  | Day 57 ^2)^ |  | Day 92 ^3)^ |
| --- | --- | --- | --- | --- | --- |
| Bilirubin, mg/dL | 2.3 |  | 0.3 |  | 0.8 |
| AST, U/L | 164 |  | 28 |  | 23 |
| ALT, U/L | 32 |  | 37 |  | 38 |
| Creatinine, mg/dL | 2.53 |  | 0.52 |  | 0.48 |
| Albumin, g/dL | 1.8 |  | 1.9 |  | 3.3 |
| BUN, mg/dL | 28 |  | 16 |  | 20 |
| BUN/Scr | 11.1 |  | 30.8 |  | 41.7 |
| Na, mmol/L | 137 |  | 149 |  | 133 |
| K, mmol/L | 8.5 |  | 3.5 |  | 4.1 |
| WBC, ×10^3^/μL | 42.1 |  | 8.1 |  | 6.2 |
| Netro, /μL | 36332 |  | 6237 |  | 4321 |
| CRP, mg/dL | 0.15 |  | 17.8 |  | 5.33 |
| PCT, ng/mL | 23.4 |  | 1.86 |  |  |
| Hemoglobin, g/dL | 20.3 |  | 9.6 |  | 8.9 |

Abbreviations: WBC, white blood cell; CRP, C-reactive protein; PCT, procalcitonin.

^1)^: At the time of hospitalization, ^2)^: At the initiation of combination therapy with tazobactam/ceftolozane and tobramycin, ^3)^: At the time of discharge form hospital.

**Table S2. Blood concentration level of tobramycin**

|  | C_min_ | C_peak_ | Dose | |
| --- | --- | --- | --- | --- |
|  | (μg/mL) | | (mg) | (mg/kg) |
| day 58 | <0.3 | － | 180 | 3 |
| day 61 | <0.3 | － | 180 | 3 |
| day 65 | <0.3 | － | 240 | 4 |
| day 69 | 0.7 | 13.1 | 270 | 4.5 |
| day 72 | <0.3 | 9.6 | 270 | 4.5 |

C_min_: Minimum blood concentration

C_peak_: Blood concentration 60 minutes after start of tobramycin administration

－: No measurement

**Table S3.** **Antimicrobial susceptibility testing of tazobactam/ceftolozane against *Pseudomonas aeruginosa***

| Organism | Days | MIC  (μg/dL) | Interpretation |
| --- | --- | --- | --- |
| *P. aeruginosa* |  |  |  |
| Sample 1 | day37 | > 8 | R |
| Sample 2 | day41 | = 8 | I |
| Sample 3 | day50 | > 8 | R |
| Sample 4 | day56 | > 8 | R |

Abbreviations: *P. aeruginosa*, *Pseudomonas aeruginosa*; MIC, minimum inhibitory concentration; R, resistant; I, intermediate.

**Table S4. Blood concentration level of amikacin**

|  | C_min_ | C_peak_ | Dose | |
| --- | --- | --- | --- | --- |
|  | (μg/mL) | | (mg) | (mg/kg) |
| day 30 | 4.4 | 25.9 | 450 | 7.5 |
| day 36 | 6.6 | － | 350 | 5.8 |
| day 42 | 2.2 | 9.3 | 150 | 2.5 |

C_min_: Minimum blood concentration

C_peak_: Blood concentration 60 minutes after start of amikacin administration

－: No measurement
